# Equine Encephalosis Virus in India, 2008

**DOI:** 10.3201/eid2405.171844

**Published:** 2018-05

**Authors:** Pragya D. Yadav, César G. Albariño, Dimpal A. Nyayanit, Lisa Guerrero, M. Harley Jenks, Prasad Sarkale, Stuart T. Nichol, Devendra T. Mourya

**Affiliations:** National Institute of Virology, Pune, India (P.D. Yadav, D.A. Nyayanit, P. Sarkale, D.T. Mourya);; Centers for Disease Control and Prevention, Atlanta, Georgia, USA (C.G. Albariño, L. Guerrero, M.H. Jenks, S.T. Nichol)

**Keywords:** equine, horse, next-generation sequencing, NGS, isolate, Reovirus, India, viruses, equine encephalosis virus

## Abstract

A virus isolated from a sick horse from India in 2008 was confirmed by next-generation sequencing analysis to be equine encephalosis virus (EEV). EEV in India is concerning because several species of *Culicoides* midge, which play a major role in EEV natural maintenance and transmission, are present in this country.

Equine encephalosis is an arthropodborne, noncontagious disease of equids, characterized by fever and lassitude (*1*). For some affected horses, lack of appetite and some degree of edema have been reported as secondary complications (*2*). Equine encephalosis virus (EEV; family *Reoviridae*, genus *Orbivirus*) was first isolated from horses in South Africa in 1967 and was considered endemic to southern Africa until an outbreak was reported in Israel in 2008 (*3*). Reports of EEV circulation in Israel indicated that the virus is not limited to a particular region and raised concerns about the risk for its spread to other countries where competent hosts and vectors are present (*3*). A recent study examining EEV seroprevalence in Israel, Palestine, and Jordan has emphasized the potential risk for invasion of pathogens into new ecologic niches (*4*).

Two species of midge, *Culicoides imicola* (species complex) and *C. bolitinos*, have been implicated as EEV vectors (*1*). Although EEV infection results in high (60%–70%) morbidity rates among equids, deaths from this infection are rare (*1*). Diagnosis of EEV infection is confirmed by several techniques, including virus isolation in baby hamster kidney cells or suckling mice and demonstration of EEV antigen by antigen capture ELISA and serum virus neutralization tests. In addition, the TaqMan minor groove binder probe reverse transcription PCR has been used for rapid detection of EEV strains and differentiation from African horse sickness virus (*5*).

The EEV genome consists of 10 double-stranded RNA segments encapsulated by a double-layered icosahedral shell. The viral genome encodes 7 structural proteins (virus capsid proteins [VPs] 1–7) and 3 nonstructural proteins (NS1–3). On the basis of cross-neutralizing antibodies, 7 distinct serotypes of EEV (EEV-1–7) have been characterized (*6*).

Recent reports of next-generation sequencing analysis assert an unbiased identification of novel viruses in clinical samples (*7*,*8*). We investigated a virus isolated from necropsy samples received in 2008 from a horse in Pune, Maharashtra, India. Data analyzed from the MiniSeq (https://sapac.illumina.com/?langsel=/in/) output led to identification and generation of the complete EEV genome.

## The Study

In July 2008, the National Institute of Virology in Pune received necropsy samples from a horse in Pune, including blood and organ tissue (lung, liver, kidney, and spleen). In addition, nasal swab, blood, and serum samples were collected from 13 horses on the same farm, reported to have clinical signs similar to those of the dead horse (fever, nasal discharge, loss of appetite, and weakness). These 13 horses recovered. No further specific details could be retrieved after this virus was identified.

All samples from the 14 horses were negative for equine influenza, Japanese encephalitis, and West Nile virus RNA according to PCR (*9*). We isolated virus by using horse tissue suspensions and blood samples as inocula. Each specimen was individually inoculated at a volume of 0.1 mL into 24-well plates containing a subconfluent monolayer of Vero CCL-81 cells. After incubating for 1 h at 37°C, the inoculum suspensions were removed and the cells rinsed twice with phosphate-buffered saline. We subsequently added Eagle minimum essential medium supplemented with 2% fetal calf serum and incubated the cells at 37°C. Postinfection cytopathic effects were observed by using an inverted light microscope (Nikon, Melville, NY, USA), and cellular morphologic changes were recorded. We determined viral titers in Vero CCL-81 cells to be 10^7.67^ 50% tissue culture infective dose/mL by using the method of Reed and Muench (*10*). We checked 8 vertebrate and invertebrate cell lines for susceptibility to this virus. Most cells, with the exception of bat embryonic cells and A549 (human lung carcinoma), displayed postinfection cytopathic effects (Table 1).

**Table 1:** Susceptibility of vertebrate and invertebrate cells to equine encephalosis virus*

Cell line	Media used	No. cell passages	CPE on PID, passage 2
Vero CCL-81	MEM	16	PID 2
Bat embryo (*Pipistrellus ceylonicus*)	DMEM	66	No CPE
PS (porcine stable kidney)	MEM	107	PID 2
BHK-21 (baby hamster kidney)	MEM + 5% TPB	83	PID 2
SW-13 (human adrenal cortex)	L-15	42	PID 2
RD (rhabdomyosarcoma)	MEM	60	PID 2
C_6_/3_6_ (*Aedes albopictus* mosquito–derived)	MM	147	PID 7
Vero E6	MEM	53	PID 2
A549 (human lung carcinoma)	Ham F-12K	86	No CPE

*CPE, cytopathic effect; DMEM, Dulbecco modified Eagle medium; MEM, Eagle minimal essential medium; PID, postinfection day; TBP, tryptose phosphate broth.

Because the initial identification attempts in 2008 yielded negative results, we performed a pathogen discovery protocol by using our recently acquired next-generation sequencing instrument. We performed DNA and RNA extractions separately on 1 mL of tissue culture supernatants or cell pellets of virus-infected cells by using a QIAamp DNA extraction kit or QIAamp Viral RNA extraction kit (QIAGEN, Valencia, CA, USA), according to manufacturer’s instructions. Concentrations of extracted RNA were quantified by using a Qubit 2.0 Fluorometer (Invitrogen, Carlsbad, CA, USA), and host ribosomal RNA was depleted by using the NEBNext rRNA Depletion Kit (New England Biolabs, Ipswich, MA, USA). We requantified this purified RNA and prepared RNA libraries by using TruSeq Stranded mRNA Library Preparation Kit (Illumina, San Diego, CA, USA). Similarly, we prepared paired-end DNA sequencing libraries from DNA samples by using a Nextera XT DNA Library Prep Kit (Illumina). We quantified these libraries by using a KAPA Library Quantification Kit (Kapa Biosystems; Roche Diagnostics Corporation, Indianapolis, IN, USA) per manufacturer’s protocol and loaded them on an Illumina Miniseq next-generation sequencing platform. We imported the raw RNA data of 229 and 170 megabytes, along with DNA data of 176 and 199 megabytes for blood and lung samples, respectively, into the CLC Genomics Workbench software (QIAGEN) for analysis.

**Figure 1:**
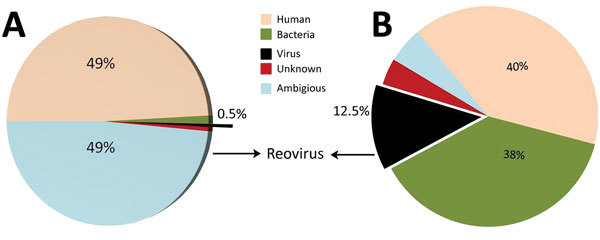
Sample identification by use of next-generation sequencing. RNA reads from blood (A) and lung (B) samples from horse that died of equine encephalosis in India, 2008. Samples were categorized by using Taxonomer software (*11*); 0.5% of the reads from blood samples (total reads 2,610,292, average length 137 bp) and 12.5% of the reads from lung samples (total reads 2,125,678, average length 135 bp) were matched to those of reoviruses.

To assemble contiguous sequences (contigs) by using de novo assembly, we used paired-end reads for DNA and RNA. DNA reads for blood and lung samples gave 166 and 113 contigs, with average lengths of 1,112 and 6,413 bp, respectively; 29 and 1,077 contigs were generated from the assembly of RNA reads for blood and lung samples, with average lengths of 1,376 and 1,034 bp, respectively. Figure 1 depicts data from the RNA reads, classified by using Taxonomer (*11*). BLAST (http://blast.ncbi.nlm.nih.gov/Blast.cgi) analysis of RNA contigs led to sample identification as EEV, and DNA contigs failed to identify any virus.

**Figure 2:**
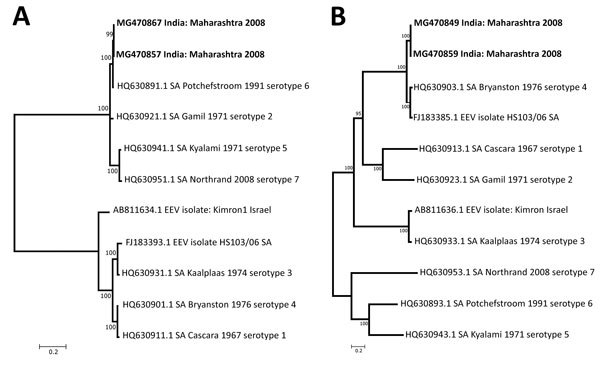
Phylogenetic tree of nonstructural 3 (A) and virus capsid protein 2 (B) genes of equine encephalosis virus. Kimura 2-parameter with (+ I) was used to create the evolutionary distance between 11 sequences of nonstructural 3 genes and virus capsid protein 2 genes from different isolates. Boldface indicates blood and lung samples from dead horse in Pune, India, 2008. GenBank accession numbers are given for reference virus sequences. Scale bars indicate nucleotide substitutions per site**.**

After the infectious agent was identified, we conducted reference sequence mapping for the RNA reads by using the EEV isolate Potchefstroom with the help of a reference-guided assembly program (CLC Genomics Workbench). We recovered the full-genome sequence of EEV by using both contigs generated from de novo assembly and the reference mapping of the reads. A total of 7 fragments encoding the structural genes VP1–7 and 3 fragments encoding the genes NS1–3, together comprising ≈19,290 nt of EEV, were generated. We compared the obtained genome sequence with other publicly available EEV sequences (Table 2; Figure 2, panels A, B). NS3 gene analysis revealed that the EEV isolate from India shared the highest identity with the Potchefstroom EEV strain from South Africa, belonging to serotype 6. However, the VP2 gene had the highest resemblance to the Bryanston EEV strain, belonging to serotype 4.

**Table 2:** Nucleotide and amino acid divergence of different EEV strains from the EEV isolated from a horse in India, 2008*

EEV GenBank sequence	Nucleotide and amino acid divergence, %																												
NS1, 1,728 nt			NS2, 1,183 nt			NS3, 769 nt			VP1, 3,948 nt			VP2, 3,158 nt			VP3, 2,758 nt			VP4, 1,958 nt			VP5, 1,584 nt			VP6, 1,062 nt			VP7, 1,165 nt	
ND	AD		ND	AD		ND	AD		ND	AD		ND	AD		ND	AD		ND	AD		ND	AD		ND	AD		ND	AD
EEV lungs NIV India	0	0		0	0		0	0		0	0		0	0		0	0		0	0		0	0		0	0		0	0
AB811632.1 EEV Kimron1	7	1		6	15		26	17		2	1		46	50		3	0		4	11		27	14		4	7		4	0
FJ183388.1 EEV HS103/06	5	2		1	5		25	16		6	1		12	8		4	1		1	3		9	2		5	8		3	0
HQ630889.1 EEV strain 6	7	2		4	12		1	2		1	0		47	55		7	1		4	10		28	17		4	6		7	0
HQ630899.1 EEV strain 4	5	2		3	7		26	15		4	1		11	8		5	0		3	8		8	1		5	9		4	0
HQ630909.1 EEV strain 1	4	2		3	10		25	15		5	1		43	47		2	0		4	11		26	12		5	7		4	0
HQ630919.1 EEV strain 2	4	1		3	9		4	4		8	2		43	48		3	0		13	32		24	11		3	5		6	0
HQ630929.1 EEV strain 3	4	2		7	19		25	15		5	1		46	50		5	0		2	6		27	15		5	8		2	0
HQ630939.1 EEV strain 5	4	2		3	7		9	7		5	1		49	57		2	0		3	8		27	16		7	10		3	0
HQ630949.1 EEV strain 7	5	2		5	13		9	7		6	1		50	59		4	0		2	4		28	17		1	2		3	0

*AD, amino acid divergence; EEV, equine encephalosis virus; ND, nucleotide divergence; NIV, National Institute of Virology, Pune, India; NS, nonstructural protein; VP, virus capsid protein.

## Conclusions

The complete genome of the EEV isolated from a dead horse confirmed the presence of EEV in India. We compared the resultant full-genome sequence of this isolate with other EEV sequences available in GenBank. Phylogenetic analysis of the VP2 gene sequences, which is used for genotype classification of reoviruses, revealed that the isolate from India groups with the Bryanston EEV strain, belonging to serotype 4.

Because no specific treatments or vaccines for EEV are available, infected horses are given supportive treatment with nonsteroidal antiinflammatory drugs. The key control measure against EEV is to restrict its spread via arthropod vectors by carefully managing horses in a stable and adhering to appropriate biosafety measures. This report of EEV in India is concerning because several species of *Culicoides* midge, which play a major role in EEV natural maintenance and transmission, are present in this country.

This information about EEV has been provided to the Department of Animal Husbandry, Dairying, and Fisheries of the Ministry of Agriculture and Farmers Welfare in New Delhi, India. The Department of Animal Husbandry should soon initiate a survey to provide information about EEV presence in equine centers and commercial farms and to screen samples from sick horses. The concern is that this virus may be widely circulating in India without having been noticed earlier.
